# The Human Intracerebral EEG Platform: A Cloud‐Based Trusted Research Environment for Collaborative SEEG Research in Epilepsy and Neuroscience

**DOI:** 10.1111/ene.70702

**Published:** 2026-08-21

**Authors:** Carolina Ciumas, Florian Perrin, Florian Sipp, Nathalie Casati, Guy Spuhler, Anthony Boyer, Jonathan Haab, Viktoria Bastic Schmid, Birgit Schaffhauser, Georges Melissargos, Evan Hancock, Olivier David, Philippe Ryvlin

**Affiliations:** ^1^ NeuroDigital@NeuroTech Lab, Department of Clinical Neurosciences Lausanne University Hospital Lausanne Switzerland; ^2^ Department of Neurology, Neurovascular Unit Orléans University Hospital Orléans France; ^3^ Biomedical Data Science Center, CHUV Lausanne Switzerland; ^4^ Aix Marseille Univ, INSERM, INS, Inst Neurosci Syst Marseille France; ^5^ Department of Pediatric Neurosurgery Fondation Lenval Nice France

**Keywords:** collaborative neuroscience research, FAIR data principles, intracerebral electroencephalography (iEEG), stereo‐electroencephalography (SEEG), trusted research environment (TRE)

## Abstract

**Introduction:**

Lack of standardised methodologies and secure processing environments for intracerebral EEG (iEEG) data creates critical barriers to international collaboration and reproducibility in clinical neuroscience. Within the framework of the Human Brain Project and EBRAINS, we have developed a specific platform, the Human Intracerebral EEG Platform (HIP), to address this challenge.

**Methods:**

The specifications considered to develop the HIP included: (1) secure access to sensitive datasets; (2) standardised data organisation following the BIDS for iEEG (BIDS‐iEEG) to ensure interoperability; (3) controlled access via a secure web‐based interface; (4) provision of software needed to analyse iEEG; (5) a structured governance model enabling institutional participation through formal data sharing agreements; and (6) alignment with FAIR principles.

**Results:**

HIP was implemented as a cloud‐based Trusted Research Environment (TRE), with a first operational release in 2023. It provides private spaces for each participating institution and researchers, as well as collaborative spaces to share data. The HIP operates many software packages, including 3DSlicer, Brainstorm, HiBoP, etc., and CiCLONE, a tool specifically designed to coregister and visualise electrodes and recording leads on MRI and brain atlas. So far, the HIP hosts 34 institutions and 10 ongoing projects, including that on heartbeat potentials that show promising findings toward a better understanding of central autonomic dysfunction in persons with epilepsy.

**Conclusions:**

The HIP addresses key barriers to large‐scale iEEG research by combining secure governance, standardised data management, and collaborative analysis within a dedicated TRE, facilitating reproducible multi‐centre research.

**Abbreviations**
AI: artificial intelligence
AI/ML: artificial intelligence/machine learning
ANS: autonomic nervous system
API: application programming interface
BIDS: Brain Imaging Data Structure
CAN: central autonomic network
CHORUS: next‐generation software stack used to implement the HIP; once fully deployed, the platform will be branded as ‘HIP powered by CHORUS’
CHUV: Lausanne University Hospital (Centre hospitalier universitaire vaudois)
DOIs: Digital Object Identifiers
DTA: Data Transfer Agreement
ECG: electrocardiogram
ECoG: electrocorticography
EZ: epileptogenic zone
GDPR: General Data Protection Regulation
GPU: graphics processing unit
HBN: Healthy Brain Network
HEP: heartbeat‐evoked potential
HFOs: high‐frequency oscillations
HIP: Human Intracerebral EEG Platform
HIPAA: Health Insurance Portability and Accountability Act
HPC: high‐performance computing
HTTPS: Hypertext Transfer Protocol Secure
iEEG: intracerebral electroencephalography
iEEG‐BIDS: intracerebral EEG Brain Imaging Data Structure
ISO/IEC 27001: International Organisation for Standardisation/International Electrotechnical Commission 27,001
MEG: magnetoencephalography
NINDS: National Institute of Neurological Disorders and Stroke
NWB: Neurodata Without Borders
PWE: people with epilepsy
SATRE: Safeguarding Trustworthy Research Environments
SEEG: stereo‐electroencephalography
SPA: Single‐Page Application
SUDEP: sudden unexpected death in epilepsy
TRE: trusted research environment
VML: Virtual Mobile Laboratory

## 1 Intracerebral EEG Research and Why It Deserves a Special Focus

Intracerebral electroencephalography (iEEG) is an invasive technique used in the presurgical evaluation of drug‐resistant focal epilepsy [1]. It is employed to delineate the epileptogenic zone (EZ) while minimising neurological and cognitive risks associated with consecutive surgical therapies [2]. iEEG is most commonly performed using stereo‐electroencephalography (SEEG), which involves the stereotactic placement of depth electrodes to enable the simultaneous recording of activity from both superficial and deep cortical structures [3]. Approximately 200–250 recording contacts are available per patient. Over the past decade, SEEG has largely replaced subdural grids in many centres due to its greater tolerability and improved access to deep brain regions such as the insula [4].

However, SEEG inherently samples only a small fraction of the brain (≤ 1%), meaning that electrodes may miss the true seizure onset zone [5]. Various interictal (e.g., high‐frequency oscillations (HFOs)) and ictal biomarkers have been proposed to address this limitation, but none have been universally validated. Due to the heterogeneity of epileptogenic pathologies, implantation strategies, resections, and outcomes, very large datasets are required to draw robust conclusions.

Beyond clinical use, SEEG provides high‐quality multimodal datasets with millimetric spatial resolution and millisecond temporal resolution, including continuous iEEG, stimulation responses, imaging and physiological recordings. As most centres perform relatively few implantations per year, large‐scale multicentre data sharing is also essential to increase statistical power and enable the development of iEEG‐based foundational models.

Large‐scale multicentre iEEG research is therefore essential, but it requires more than data aggregation alone: It also depends on secure governance, standardised data organisation, reproducible analytical workflows, and collaborative environments adapted to sensitive clinical datasets. The Human Intracerebral EEG Platform (HIP) was developed within the Human Brain Project and EBRAINS [6] to address these needs within a dedicated, trusted research environment (TRE). In this review, we examine the current landscape of iEEG data‐sharing solutions, outline the relevance of cloud‐based TREs for iEEG research, describe the design and objectives of the HIP, and illustrate its scientific value through a translational use‐case on heartbeat‐evoked potentials. We then discuss the main limitations, sustainability challenges, and future directions for large‐scale iEEG research.

## 2 Existing iEEG Data‐Sharing Resources, Standards and Remaining Gaps

Several initiatives have addressed the challenge of organising and disseminating electrophysiological data, including intracranial EEG (iEEG), though each offers only partial coverage of the needs specific to clinical and research applications.

One such initiative is *Neurodata Without Borders* (NWB) [7], which has established an open‐source data standard to harmonise the storage and analysis of neurophysiology recordings, including intracellular and extracellular recordings, as well as iEEG. NWB is primarily a data standard rather than a repository. Within HIP, NWB is considered complementary to iEEG‐BIDS, and interoperability can be achieved through existing conversion tools and hybrid approaches embedding NWB files within BIDS datasets. NWB has enabled researchers to build compatible analysis tools and ensure interoperability across datasets by providing a unified standard.

EEGBase was developed as a complementary platform focused on storing, annotating, and analysing EEG data [8]. Although *EEGBase* is primarily used for scalp EEG, it has also supported the integration of iEEG recordings, thereby facilitating collaborative research across institutions. A handful of repositories provide partial solutions, but most are constrained in terms of scope, accessibility, or sustainability.

ieeg.org, e.g., is a collaborative initiative funded by the U.S. National Institute of Neurological Disorders and Stroke (NINDS) and has become a major reference point for sharing iEEG datasets, particularly those from grid electrode studies [9]. At the time of submitting this manuscript, ieeg.org was hosting 995 datasets, which were freely available upon registration, along with integrated tools for cloud‐based analysis.

A European initiative, the EPILEPSIAE database, was developed to provide annotated long‐term EEG recordings from over 250 patients [10]. The database includes datasets with recordings acquired using up to 122 channels. The project aimed to support research into seizure detection and prediction by providing standardised datasets and shared analytical resources. Access to the database is not open, but a paid licence is available for research purposes at a reduced cost. Substantially higher fees apply for commercial use.

Other platforms demonstrate alternative approaches. Several additional tools provide solutions but do not directly address the combined clinical, regulatory, and multimodal needs of modern iEEG research.

*The Virtual Mobile Laboratory* (VML) is a collaborative tool designed for the remote review and management of multimodal clinical data, including EEG, MRI, and CT scans [11]. Although powerful, access to the VML is restricted to paying users, which limits its potential to promote open science. Similarly, the *Healthy Brain Network* (HBN) provides a large‐scale, unique repository of multimodal data, including EEG, MRI, and eye‐tracking data, from approximately 1000 individuals [12]. While the HBN is freely available, its scope is developmental psychiatry rather than epilepsy. In contrast, the Montreal Neurological Institute's *Open iEEG Atlas* offers curated intracranial EEG data from multiple centres, including resting‐state and sleep recordings from over 100 subjects [13]. This initiative provides a valuable normative reference for iEEG signals but is limited in terms of underlying pathologies. In the United States, the Computational Memory Lab at the University of Pennsylvania has made large ECoG datasets collected during memory experiments available, particularly through the “Restoring Active Memory” project [14]. While these datasets are invaluable for cognitive neuroscience, they are task‐specific and restricted to certain electrode types and implantation strategies.

### 2.1 Fragmentation and Gaps in Existing Solutions

Across platforms, persistent shortcomings include:

- Lack of unified data formats and metadata standards, hindering reproducibility and cross‐dataset analyses [15].
- Variable access restrictions, from institutional barriers to subscription models.
- Insufficient multimodal integration, particularly regarding MRI/CT coregistration and surgical annotations.
- Absence of GDPR‐compliant frameworks in US‐centric resources, limiting transatlantic data sharing: A major hurdle for trans‐Atlantic iEEG data sharing is that the EU's GDPR treats neuroimaging and electrophysiology data as inherently non‐anonymizable and therefore requires strict, explicit‐consent, privacy‐preserving controls, while the US relies on a far more permissive, fragmented opt‐out model—creating a fundamental regulatory incompatibility.

Taken together, existing resources offer valuable, albeit incomplete, solutions. Some focus on data standards, some on data availability, and some on specific research domains or electrode configurations. However, persistent limitations remain across the current landscape in the areas of secure governance for sensitive clinical data, multimodal integration with imaging and surgical annotations, reproducible cross‐centre workflows, and compatibility with European regulatory requirements for data sharing. The HIP is not intended to replace these resources, but rather to complement them by providing a governed Trusted Research Environment (TRE) for sensitive clinical iEEG and multimodal data. Interoperability with existing repositories and standards is facilitated through iEEG‐BIDS, metadata harmonisation, and emerging interoperability with NWB. Ongoing activities within EBRAINS, EOSC‐related initiatives, and the emerging European Health Data Space (EHDS) are expected to further strengthen these connections.

## 3 Cloud‐Based Trusted Research Environments (TREs) and Their Importance for SEEG‐Based Research

Cloud‐based TREs provide secure, ethical, and technically robust frameworks which facilitate data sharing and collaboration [16]. They are designed to manage sensitive (health) data while maximising its research potential by enabling access and analysis without compromising privacy, security, or the rights of data owners. This balance is achieved through a governance framework known as the “Five Safes”: safe data (appropriately de‐identified and curated); safe projects (scientifically and ethically approved); safe people (accredited researchers); safe settings (controlled digital environments); and safe outputs (results checked for disclosure risk before release) [17]. Together, these pillars ensure the integrity of sensitive datasets from ingestion to dissemination. By enforcing data protection by design, standardising data formats, and embedding governance mechanisms, TREs provide the necessary legal framework and ethical safeguards for the reuse of sensitive data.

TRE adoption has accelerated rapidly across domains such as genomics, oncology, and population health. Large‐scale national initiatives in the United Kingdom, the United States, and the European Union have adopted TRE‐based infrastructures as the foundation for collaborative science, ensuring compliance with stringent data protection regulations such as the General Data Protection Regulation (GDPR) in Europe and the Health Insurance Portability and Accountability Act (HIPAA) in the US. Although HIP has been designed primarily within a GDPR‐compliant European framework, its governance model can support international collaborations. Data contributions from non‐European jurisdictions are assessed on a project‐specific basis through appropriate legal agreements, including Data Transfer Agreements (DTAs) and Data Sharing Agreements (DSAs), ensuring compliance with both local and European regulatory requirements. These precedents demonstrate not only scalability but also long‐term sustainability. This is because they rely on sustained institutional support, federated architectures, and continuous integration into national research ecosystems.

Beyond governance, TREs can be particularly powerful due to their ability to integrate secure data repositories with high‐performance computing (HPC) resources and cloud‐based GPU clusters. This architecture, combining secure infrastructure with secure compute and processing environments, is essential for applying modern deep‐learning methods, including foundational models, to sensitive data. Such a system is now being implemented within the evolving HIP infrastructure and is especially important for computationally intensive iEEG time series, which can reach up to 2 TB for a single patient. By uniting strong data security with advanced computational capabilities, cloud‐based TREs create a scalable environment that enables iEEG research to produce reproducible and clinically meaningful discoveries.

For SEEG centres, a TRE such as the HIP offers additional advantages, including seamless access to a wide range of applications and software tools for analysis, as well as validated analysis pipelines. Moreover, it provides strict control over data usage to reassure data owners, and a collaborative ecosystem in which projects can be launched across institutions while preserving local ownership and oversight. Thus, TREs enable not only secure and scalable iEEG data sharing but also foster trust, reproducibility, and innovation within the clinical and research community.

A fundamental function of any TRE is the enforcement of robust data management, legal, and ethical safeguards to protect both patients and contributing institutions. This includes strict governance structures that define rights, responsibilities, and acceptable use, supported by formal agreements governing data transfer, storage, and reuse. Within the HIP, this is implemented through mandatory Data Transfer Agreements (DTAs), which specify data ownership, contributor obligations, consent requirements, and privacy‐preserving measures. These mechanisms ensure that data sharing remains secure, ethically compliant, and aligned with European regulatory standards while enabling impactful, large‐scale collaborative research.

Access to collaborative projects is governed through accreditation of authorised users, project approval procedures, and DTAs defining ownership, permitted use, consent restrictions, and output‐sharing conditions, consistent with the Five Safes framework.

## 4 The Human Intracerebral EEG Platform (HIP) and Why It Stands Out in the Context of iEEG‐Driven Research

### 4.1 HIP History and Objectives

The HIP benefits from strong institutional backing. It is formally supported by the European Reference Network EpiCARE, which comprises around 50 epilepsy centres across Europe. Many of these centres routinely perform iEEG investigations in cases of complex and rare epilepsy. Discussions are underway to expand participation to other continents. The governance structure is formalised through a charter that clearly defines the roles and responsibilities of data controllers, contributors and users. Project entry into collaborative workspaces requires approval under predefined governance procedures. User access is granted according to project membership and accredited researcher status and may be modified or revoked when project participation changes. This ensures trust, accountability and adherence to the highest ethical standards.

While structured clinical metadata and semiology annotations can be shared within the platform, raw patient video recordings are considered particularly sensitive and therefore remain under local centre responsibility. Alternative approaches include the use of structured semiology annotations, time‐stamped event descriptors, and derived non‐identifying metadata sufficient for research analyses. Emerging privacy‐preserving technologies, including automated face‐swapping and video de‐identification approaches, may provide additional opportunities for secure sharing of video‐derived information in the future, subject to appropriate governance, consent, and regulatory requirements.

### 4.2 HIP Architecture

**FIGURE 1:**
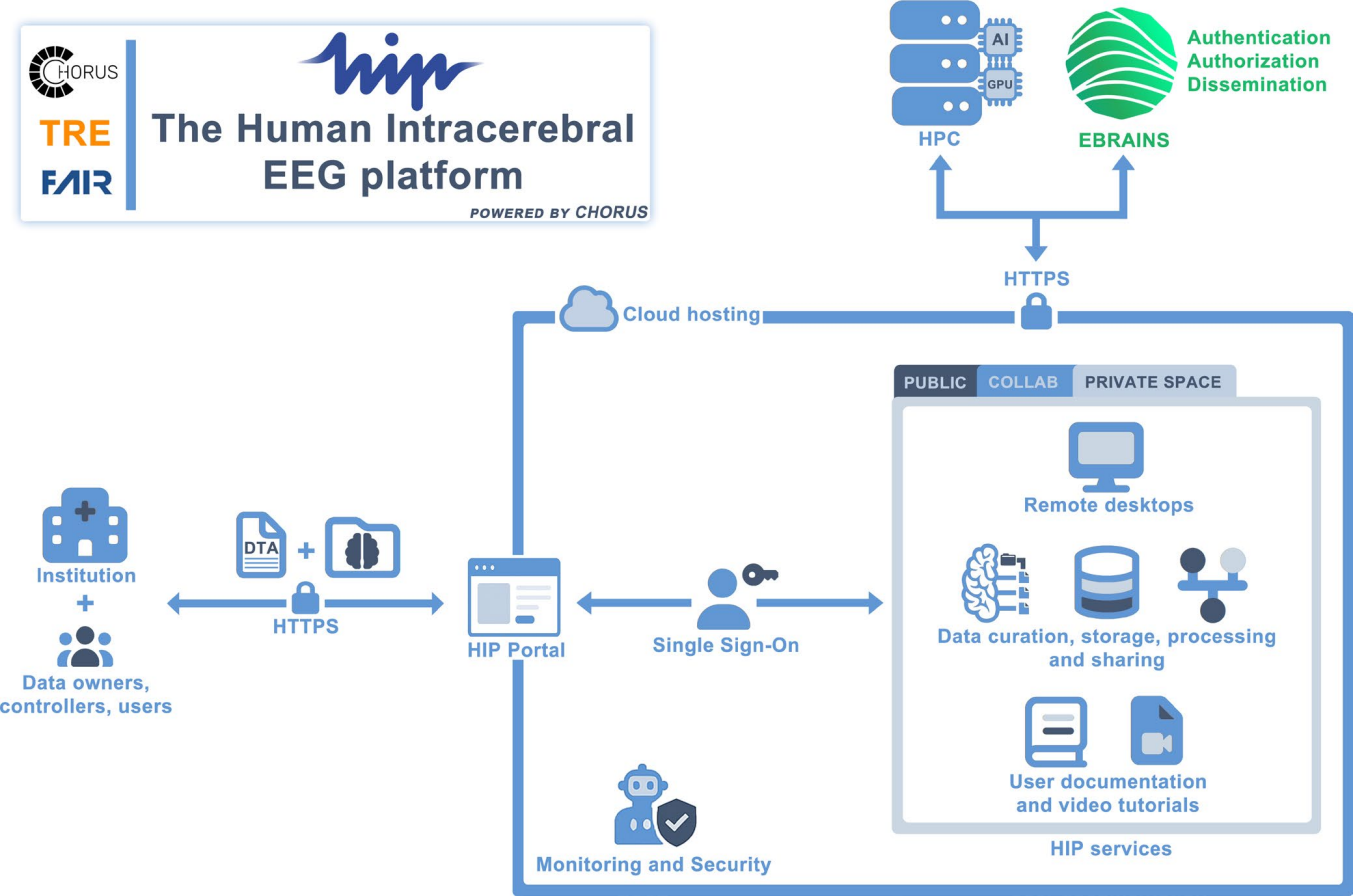
Overview of the HIP. The HIP is a secure Trusted Research Environment (TRE) designed for the management, processing, and sharing of intracerebral EEG and multimodal neuroscience data. Data are transferred from participating institutions to the HIP Portal via secure HTTPS connections with controlled access. HIP provides private, collaborative, and public workspaces, as well as remote computing environments and data curation and storage services. It also integrates with EBRAINS and HPC infrastructures to enable advanced analysis and dissemination. Continuous monitoring, authentication, and security mechanisms ensure compliant, FAIR data management.

The HIP is currently organised around a three‐tier model designed to balance confidentiality with scientific openness (Figure 1).

- Personal space: A secure, access‐controlled environment in which each individual data provider can upload, curate, and process its own pseudonymised datasets, while no other HIP user can get access to such space. Storage allocation is managed through project‐level quotas and infrastructure monitoring, with extensions requiring technical and governance approval.
- Collaborative space: An access‐restricted environment that allows multicentre teams to share curated datasets and run analyses jointly on previously approved research projects.
- Public space: The future HIP roadmap includes the creation of a fully anonymised environment in which authorised datasets are made openly available through the EBRAINS Knowledge Graph. These datasets will be assigned persistent Digital Object Identifiers (DOIs) and enriched with metadata to facilitate findability, interoperability, and reuse. Transfer of datasets from collaborative projects to the public space is only possible when explicitly authorised by the relevant consent framework and corresponding DTA/DSA provisions.

This architecture enables data controllers to retain oversight of their contributions while supporting collaborative projects and promoting open science. These design principles are essential and preserved in the platform's current software evolution.

The HIP frontend is engineered as a modern Single‐Page Application (SPA) using React and TypeScript, providing neuroscientists with a secure and intuitive graphical interface that abstracts the complexities of the underlying cloud infrastructure. The user experience is organised around a dual workspace paradigm “Center” (private institutional) and “Project” (collaborative) workspaces, managed via client‐side routing and a centralised state management store. This model mirrors real‐world research dynamics and mitigates accidental data exposure.

From a technical standpoint, the frontend interacts with the backend exclusively through a dedicated API client layer. Every user action—from browsing files to launching an application—is translated into a secure, authenticated HTTPS request to the API Gateway, with user identity verified via session tokens on each call. This ensures that all operations are authorised against the robust access control policies defined by the user's workspace memberships.

The HIP is deployed on a secure cloud infrastructure certified under ISO 27001, ISO 27017 and ISO 27018, approved for French Health Data hosting (HDS), and compliant with GDPR and HIPAA requirements for the handling of sensitive data. This infrastructure also supports the platform's ongoing software evolution.

The HIP is currently being migrated from its initial VM‐based implementation to a fully Kubernetes‐orchestrated software platform that encompasses the frontend, backend, and associated services. This transition substantially improves the platform's scalability, resilience, and maintainability. The new implementation relies on a multi‐machine architecture using Kubernetes‐managed clusters, allowing computational resources to be allocated according to workload requirements. For more demanding GPU‐intensive tasks, including the training or inference of foundation models for neuroscience, the HIP is being designed to support access to established AI supercomputing centres through dedicated architectural blueprints.

At the time of writing, HIP supports multiple participating centres and several ongoing collaborative projects. Implemented functionalities include secure workspaces, BIDS‐based data curation workflows, integrated software environments, and controlled multicentre data sharing, while some roadmap elements described later remain under active development.

### 4.3 HIP Software Ecosystem and Data Processing

The HIP provides access to a rich ecosystem of widely adopted open‐source software tools for the formatting, analysis and visualisation of iEEG data (Bidsificator [18], TRCanonymiser [19], Brainstorm [20], Intranat [21], HiBoP [22]) and associated neuroimaging (NiftyReg [23], FreeSurfer [24], 3D‐slicer [25], MRIcroGL [26]). It also operates generic software (Matlab [27] RStudio [28] VSCode [29]) and dedicated iEEG pipelines such as CiCLONE for depth electrodes' localisation [30]. Unless otherwise specified, these applications are provided for research purposes and should not be considered certified clinical decision‐support systems. Responsibility for their use remains with the user and their institution in accordance with applicable licences, DTAs and local governance requirements. Other open‐source tools and pipelines are expected to be integrated in the future based on community requirements, particularly those dedicated to AI‐based iEEG data curation and analysis.

The HIP provides a suite of integrated tools accessible via a web browser, enabling the seamless use of applications originally designed for native desktop environments. This approach offers significant benefits, including standardised data processing and centralised maintenance and upgrading of software and dependencies. The HIP secure processing environment enables clinicians and researchers to access secure virtual workspaces from any location, including directly from within hospital environments, thus avoiding the need for complex software installation on local machines and reliance on the limited computational resources of individual devices. This design lowers the barrier to adoption, enabling users with minimal coding expertise to process iEEG data within a controlled, privacy‐preserving infrastructure. Integration of commercial or industry‐supported software may be considered in the future, if licencing, interoperability, cybersecurity and regulatory requirements remain compatible with the TRE framework.

### 4.4 The HIP Data Management

To ensure interoperability and reproducibility, all SEEG, MRI, and CT datasets contributed to the HIP adhere to the iEEG‐Brain Imaging Data Structure (iEEG‐BIDS) standard. Initial pseudonymisation and preparation are performed locally by the contributing centre before upload into the HIP private space. BIDS conversion and curation can then be performed using dedicated HIP tools, including the Bidsificator. Automated validation procedures are integrated into the workflow, and only curated datasets approved by the contributing centre may subsequently be shared within collaborative projects. Data management relies on a distributed storage architecture optimised for large‐scale data streaming. This approach enables selective access to specific segments of large files without requiring full data transfer. Such functionality is particularly critical for long‐duration iEEG recordings, allowing clinicians to rapidly interrogate targeted temporal segments for diagnostic purposes, even when handling multi‐gigabyte datasets. Although iEEG‐BIDS remains the primary organisational framework within HIP, interoperability with NWB is supported through existing conversion tools and hybrid approaches, providing flexibility for different neurophysiology workflows.

## 5 The HIP in Action—A Translational Use‐Case: Heartbeat‐Evoked Potentials

Several collaborative iEEG research projects are ongoing on the HIP, including one focusing on heartbeat‐evoked potentials (HEPs), detailed below. The HEP project aims to further explore the physiology of the central autonomic network [31] (CAN) as well as its dysfunction in epilepsy, which may contribute to sudden unexpected death in epilepsy patients (SUDEP).

The CAN orchestrates the regulation of the autonomic nervous system (ANS) and its sympathetic and parasympathetic divisions that control vital bodily functions, including cardiac, respiratory, gastric, and glandular activity. It is continuously modulated by visceral signals from the ANS and integrates cardiac and respiratory control through subcortical and cortical hubs, including the insula, cingulate cortex, amygdala, and hypothalamus [31].

One way to investigate the dynamics of the CAN is to examine how bodily signals are integrated within this network. In the context of cardiac function, a widely used non‐invasive electrophysiological marker is the HEP [32, 33], defined as the cortical response time‐locked to the R‐wave of the electrocardiogram (ECG). This neural response is thought to reflect cortical processing of cardiac activity and may provide valuable insights into the functioning of the CAN, as well as into autonomic dysfunction observed in epilepsy and other disorders. Indeed, abnormal scalp‐recorded HEPs have been reported in PWE.

The broader study of bidirectional brain–body communication through HEPs has attracted increasing interest, reflecting growing recognition that the processing of visceral signals is a fundamental component of cognitive function and conscious experience [33, 34]. Accumulating evidence suggests that it influences various aspects of cognition, including perception, emotion, and interoception [34, 35, 36, 37, 38, 39].

However, the study of HEPs is significantly hindered by methodological limitations. MRI lacks the temporal resolution required to capture the fast neural dynamics of heartbeat processing, while scalp EEG, despite its temporal precision, suffers from poor spatial resolution and source localisation challenges. In addition, HEPs are often contaminated by cardiac‐related artefacts, such as the cardiac field artefact arising from the heart's electrical activity and the pulse artefact related to brain tissue motion [33, 34]. The lack of standardised and effective correction methods has led to inconsistent and sometimes non‐replicable findings in scalp EEG research [33, 34]. In this context, iEEG emerges as an optimal approach, providing continuous recordings of both ECG and cortical regions that are often inaccessible with scalp EEG, particularly the insula, cingulate cortex, and amygdala.

In parallel, HEPs have recently been proposed as potential electrophysiological markers of residual consciousness in patients with disorders of consciousness [40]. Despite this growing interest, HEP remains largely understudied during altered states of consciousness such as sleep. Previous work has relied almost exclusively on scalp EEG, with inconsistent findings regarding vigilance‐related modulation and has mainly focused on sleep disorders or REM sleep [41, 42].

To date, only a few studies have investigated HEPs using iEEG data. These studies have so far concentrated on wakefulness [43, 44, 45] and, more rarely, REM sleep [46]. Grid‐based recordings predominantly identified responses in the somatosensory cortex, whereas depth electrodes revealed HEP‐related activity in the insula, opercular cortex, inferior frontal gyrus, amygdala, and thalamus. Despite these promising initial findings, research in this area remains limited by the scarcity of available data and the small number of patients included. Pooling HEP datasets from different centres via the HIP therefore represents a crucial step toward advancing our understanding of brain–heart interactions and the CAN in epilepsy and related conditions.

Here, we conducted the first systematic intracranial exploration of HEPs across vigilance states, with a specific focus on the insular cortex as a key interoceptive hub of the central autonomic network. In the first phase, 10 patients were analysed using pseudonymized datasets stored in the HIP private space. For each patient, one seizure‐free night of continuous recording (approximately 8–12 h) was screened to identify suitable periods containing roughly one hour each of wakefulness, N2 sleep, and N3 sleep. Thanks to the platform's centralised storage and computing resources, native sampling rates (1024 Hz) were preserved throughout preprocessing, allowing full exploitation of SEEG temporal precision. Preprocessing and statistical analyses were conducted using the HIP software ecosystem, notably Brainstorm and MATLAB with specialised toolboxes. HEP responsiveness was assessed using computationally intensive surrogate‐based procedures requiring large numbers of permutations, made feasible by the HIP's delegated computing environment.

**FIGURE 2:**
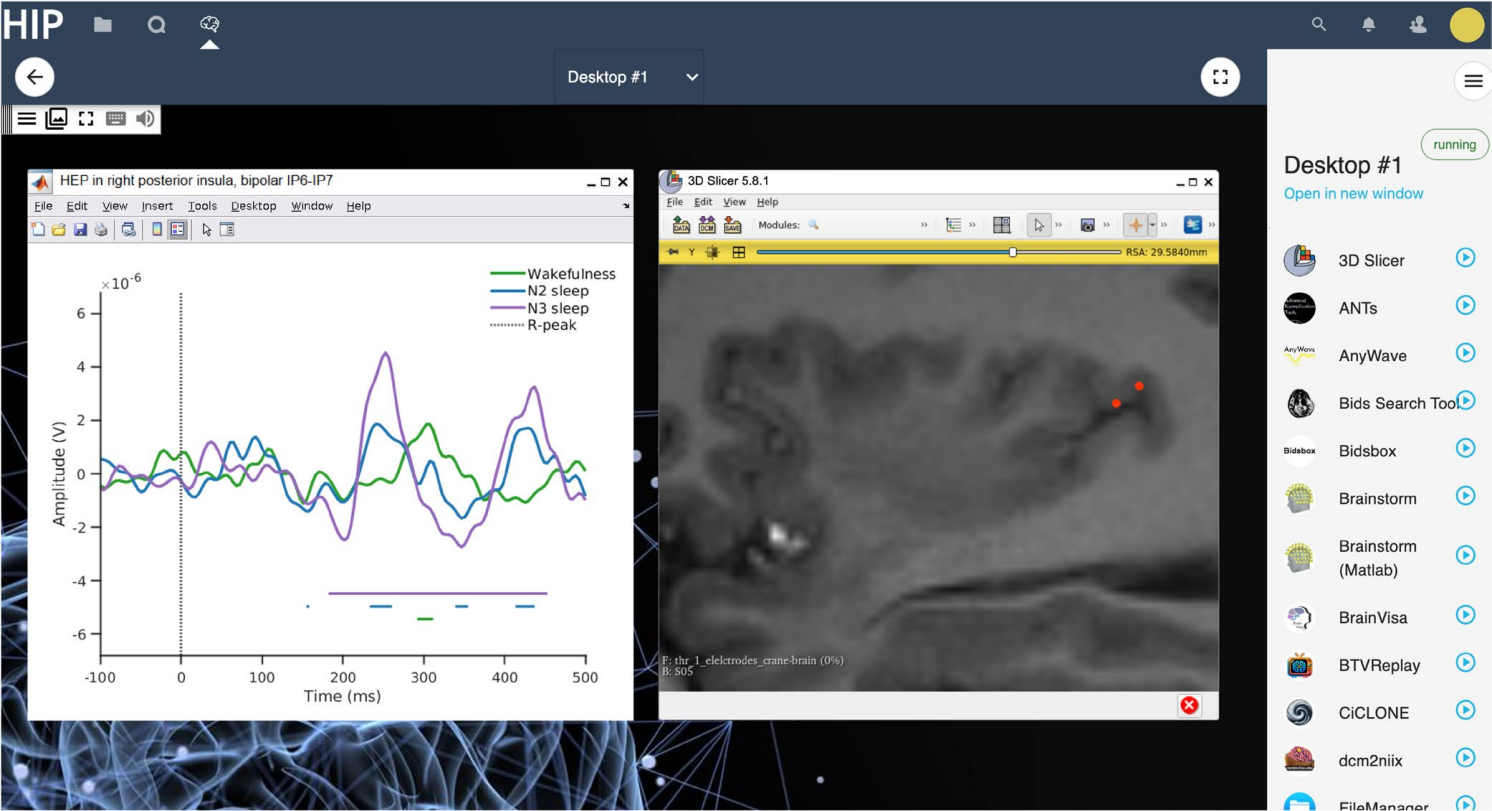
HIP user workspace and analysis environment. Example of a HIP remote desktop session showing integrated neuroscience applications within a secure, cloud‐based workspace. Users can access neuroimaging and electrophysiology software (e.g., 3D Slicer, ANTs, AnyWave and Brainstorm), as well as data visualisation and analysis tools. This enables the collaborative processing of intracerebral EEG and multimodal datasets without the need to download local data.

From 1858 implanted contacts, 256 bipolar grey‐matter contacts within predefined CAN‐related regions were retained after anatomical selection and artefact rejection; 55 (21.4%) were HEP‐responsive in at least one vigilance state. The posterior insular cortex showed the highest density of responsive sites, with additional responses in somatosensory and motor cortices and sparser involvement of associative regions. The influence of heart rate on HEP topography was not specifically investigated in the present study because the available sample size was insufficient to robustly evaluate heart‐rate‐dependent spatial effects across insular subregions. Importantly, HEPs were observed during both wakefulness and NREM sleep. Focusing on posterior insular contacts, mixed‐effects modelling revealed a significant enhancement of HEP amplitude during deep sleep (N3) compared with both wakefulness and N2 (Figure 2). This effect remained significant after controlling for spike rate, used as a proxy for local epileptiform activity, arguing against a trivial explanation whereby the larger amplitudes observed during N3 could be driven by spike‐related signal contamination, known to be more prevalent during sleep. In fact, higher spike rates were associated with lower HEP amplitudes, suggesting a possible attenuation of physiological cardiac signal integration in contacts with greater epileptiform activity. Although this observation requires confirmation in larger samples, it raises the possibility that network hyperexcitability may interfere with interoceptive processing in epilepsy.

The project has now entered its second phase with the inclusion of 10 additional patients in the HIP collaborative space, coming from an independent SEEG centre. The objective is to progressively scale this effort toward a large multicentre cohort, potentially reaching an order of hundreds of patients, thereby overcoming the traditional limitation of small sample sizes in SEEG research. This expansion will enable a sufficiently powered dataset to characterise HEP dynamics across the full central autonomic network, examine how these responses vary across vigilance states, and compare regions participating in or outside the epileptogenic network, ultimately shedding light on the mechanisms underlying autonomic dysfunction in epilepsy and related conditions.

Beyond epilepsy, the HEP paradigm demonstrates how the HIP can serve as a model for brain–body neuroscience with translational relevance across domains such as psychiatry (e.g., anxiety and panic disorders, where altered interoception is a symptom), neurosurgery (e.g., autonomic risk mapping during intervention), and consciousness research. The HIP's secure data infrastructure, multicentre scale, and advanced analytics pipelines position it as a strong platform for further work on interoception and its clinical implications.

## 6 Limitations and Sustainability Challenges

Operating and maintaining a TRE for sensitive iEEG and multimodal neuroimaging data incurs significant ongoing financial efforts [47]. Beyond infrastructure deployment, long‐term sustainability requires continuous investment in secure hosting, high‐performance computing resources (including GPU‐enabled servers), system maintenance, regulatory compliance, cybersecurity monitoring, and dedicated technical personnel. These structural costs are rarely covered by conventional competitive research grants, which are usually short‐term and focused on project‐specific expenses.

Ensuring long‐term sustainability requires commitment from multiple sides, going beyond short‐term, grant‐based funding. In this context, EBRAINS provides a well‐established European research infrastructure and a stable framework for secure data sharing, including services for data storage, identity management, and interoperability. Such infrastructure is essential to ensure operational continuity and compliance with regulatory standards over time. Importantly, sustained financial support must also be secured at the national and institutional levels. This may include future support through national infrastructures, institutional commitments, research grants, and complementary cost‐recovery mechanisms or, in the future, a mixed sustainability model combining institutional support, national and international infrastructure funding, project‐based grants, and selective cost‐recovery mechanisms for resource‐intensive services—particularly for resource‐intensive components such as secure data storage and high‐performance computing. The ongoing migration of HIP's software stack to CHORUS, together with CHUV's support, represents an important contribution toward ensuring the platform's long‐term operational stability and viability.

Beyond financial sustainability, regulatory and operational constraints remain major challenges. DTAs between institutions often involve lengthy legal negotiations, delaying data integration and hindering the rapid scaling of multicentre datasets. Greater regulatory alignment, including the adoption of standardised agreement templates and interoperable legal frameworks across countries, is therefore essential to streamline collaboration and reduce administrative burden. In this context, a European digital research infrastructure such as EBRAINS can play a crucial enabling and facilitating role. Importantly, HIP sustainability differs from that of conventional repositories because a TRE must additionally support secure processing environments, governance workflows, fine‐grained access control, and compliance with certification standards. Interoperability with external repositories may also reduce unnecessary duplication of large datasets and associated storage costs.

In parallel, robust anonymisation of iEEG data presents specific technical and ethical complexities. While the signals themselves are not directly identifiable, associated metadata, imaging data, and clinical variables may introduce risks of re‐identification. Addressing these challenges requires the implementation of validated, standardised protocols for anonymisation and pseudonymisation that are consistently applied across centres. Such approaches can facilitate GDPR‐compliant data sharing while preserving scientific utility and reducing duplication of effort in local governance processes.

Overall, harmonising regulatory frameworks, standardising data governance procedures, and adopting shared protocols are critical to making cross‐border data sharing more efficient, less bureaucratic, and scalable. These efforts are essential to ensure that large‐scale iEEG data infrastructures remain sustainable, compliant, and scientifically impactful over the long term.

## 7 Future Directions

The next stage of HIP development is expected to broaden the platform's scientific applications and technical capabilities in several areas. Several key developments can be anticipated:

### 7.1 Artificial Intelligence and Foundational Models

AI is particularly well suited for addressing the complexity, volume, and heterogeneity of iEEG time series, and is increasingly used to automate detection and characterisation of epileptic transients (e.g., spikes and high‐frequency oscillations such as ripples and fast‐ripples) and to improve discrimination between physiological vs. pathological events, an important step for robust identification of epileptogenic tissue while preserving non‐epileptic brain regions for the study of normal function. These directions are directly relevant both for biomarker discovery and for improving delineation of the EZ in drug‐resistant epilepsy.

A particularly promising and emerging research direction is to develop foundation models (FMs) that learn reusable representations from large‐scale, multi‐centre intracranial recordings, enabling downstream decoding of cognitive functions (e.g., speech‐ and perception‐related neural signatures) and supporting more precise EZ‐related inference tasks under standardised evaluation protocols. The feasibility of this paradigm is illustrated by BrainBERT [48], which adapts mask‐modelling transformers to intracranial data but was pretrained on a comparatively limited corpus reported as 43.7 h across 10 subjects (4,551 electrode‐hours; 1249 electrodes used after preprocessing/quality screening). The HIP will serve as the backbone for training next‐generation epilepsy and neuroscience FMs by (i) enabling multi‐site dataset curation and harmonisation using established standards such as EEG‐BIDS/iEEG‐BIDS and BIDS‐compatible tooling, (ii) supporting parallel, multi‐tenant development by multiple research teams with strong isolation controls, and (iii) exploiting elastic access to high‐performance GPU resources for computationally intensive pretraining, training, and systematic ablation of design choices (e.g., tokenisation schemes, embedding interfaces, and attention mechanisms). In doing so, HIP will make it practical to move from single‐dataset FM efforts toward larger, multi‐centre and ultimately multi‐modal model development within a governed “data stays in the environment” paradigm typical of TREs, supporting a credible pathway toward a more generalised model where appropriate.

### 7.2 Multimodal Integration

While the HIP was designed specifically to manage iEEG data, its infrastructure and functionalities allow for the incorporation of many other sources of Human brain data, such as those provided by neuroimaging (already being used for localising iEEG recording contacts), scalp‐EEG, MEG, and wearable devices. Accordingly, the HIP has been used to organise a challenge in scalp‐EEG‐based seizure detection. These various sources of information could further enrich foundational models of brain dynamics.

### 7.3 Expansion Beyond Epilepsy

Although epilepsy remains the platform's primary clinical focus, the underlying infrastructure is also relevant to other domains that generate sensitive intracranial or multimodal brain data. Potential future applications include psychiatry, neurosurgery, brain–computer interface research, deep brain stimulation studies, and systems neuroscience. Any such expansion should remain driven by clearly defined scientific and governance needs rather than by platform breadth alone.

### 7.4 Deployment and the International Federation of HIP

Designed as a centralised TRE for iEEG data, HIP is now evolving toward cross‐institutional deployment as a federated infrastructure. Although centralised storage remains an option, HIP supports distributed, interoperable installations. This enables SEEG centres to retain local or national data hosting in accordance with institutional and regulatory requirements. Several European countries and the United States are undertaking federated deployment initiatives, reflecting the platform's ambition to operate as a scalable international infrastructure rather than a single‐site solution.

### 7.5 Alignment With Evolving TRE Standards

The definition and requirements of TREs continue to evolve. The most detailed consensus framework is currently provided by the Safeguarding Trustworthy Research Environments (SATRE) specification [49]. The latest stable release (SATRE v1.1.0, 18 August 2025) defines concrete technical and organisational safeguards, including strict controls on data extraction and user interaction within secure environments. These safeguards are planned within HIP's software architecture.

The forthcoming SATRE v2.0 specification is expected to expand the requirements to include cross‐site federation and the secure release of artificial intelligence/machine learning (AI/ML)‐derived outputs [50]. In anticipation of these developments, the new HIP implementation has been designed with federation‐ready infrastructure and a roadmap that addresses advanced computing security and data governance controls.

This positions HIP as an operational, forward‐looking framework rather than a static implementation: It is a sustained, standards‐aligned, future‐proof TRE infrastructure.

## 8 Conclusion

The Human Intracerebral EEG Platform was developed in response to a central challenge in epilepsy and clinical neuroscience: How to enable secure, standardised and reproducible multicentre research on sensitive iEEG datasets. By combining trusted research environment governance, BIDS‐based data organisation, collaborative workspaces and integrated analytical tools, the HIP provides a practical framework for large‐scale SEEG research across institutions.

The platform's value lies not only in data storage and access control, but also in its ability to support shared workflows and computationally demanding analyses within a controlled environment. The heartbeat‐evoked potential project illustrates how such an infrastructure can enable translational neuroscience questions that would be difficult to address within isolated single‐centre datasets.

Although important regulatory, organisational, and sustainability challenges remain, the HIP represents a concrete step toward more collaborative and reproducible iEEG research. More broadly, it offers a model for how dedicated TREs can support sensitive multimodal neuroscience data and may help accelerate biomarker validation, methodological standardisation, and cross‐centre discovery in epilepsy and beyond.

## Author Contributions

**Nathalie Casati:** methodology, validation, software, data curation. **Jonathan Haab:** methodology, validation, software, data curation. **Guy Spuhler:** methodology, validation, software, data curation. **Georges Melissargos:** methodology, validation, software, data curation, writing – review and editing. **Florian Perrin:** formal analysis, data curation, software, methodology, validation, visualization, writing – original draft. **Olivier David:** writing – review and editing, validation, software. **Viktoria Bastic Schmid:** resources, project administration. **Anthony Boyer:** methodology, validation, software, data curation, visualization. **Birgit Schaffhauser:** project administration, resources, writing – review and editing, funding acquisition. **Evan Hancock:** visualization. **Philippe Ryvlin:** conceptualization, investigation, funding acquisition, writing – original draft, writing – review and editing, validation, visualization, project administration, supervision, resources. **Carolina Ciumas:** conceptualization, investigation, writing – original draft, methodology, validation, writing – review and editing, software, project administration, supervision, resources, formal analysis. **Florian Sipp:** methodology, validation, software, data curation.

## Funding

This work was co‐funded by H2020‐SGA‐FETFLAG‐HBP‐2019, Specific Agreement No. 945539 (Human Brain Project SGA3), and by HORIZON‐INFRA‐2022‐SERV‐B‐01, Project No. 101147319 (EBRAINS 2.0). OD and AB were additionally supported by the Agence Nationale de la Recherche (ANR; grant nos. ANR‐21‐NEUC‐0005‐01, ANR‐22‐CE17‐0057‐03, ANR‐22‐PESN‐0012, and ANR‐25‐CE37‐2927‐01).

## Ethics Statement

The HEP use‐case analyses were performed on pseudonymised datasets collected under locally approved clinical and research protocols at the participating institutions. All patients provided informed consent according to local regulations. Data sharing and analysis within the HEP were conducted in accordance with applicable ethical, legal, and data protection requirements.

## Conflicts of Interest

The authors declare no competing financial interests. Several authors are involved in the development, governance, and scientific coordination of the HIP, which is the subject of this review.

## Data Availability

The datasets analysed during the HEP use‐case study are not publicly available as they contain sensitive intracerebral EEG recordings that are subject to institutional, ethical and legal restrictions. However, access to pseudonymised data may be granted via the HIP, subject to approval by the relevant data controllers, ethics committees, and data‐sharing agreements. Information regarding access procedures is available through the HIP governance framework, which is described in this manuscript.
